# *Staphylococcus aureus* nasal carriage and bloodstream infection among conventional hemodialysis patients in Thailand: a prospective multicenter cohort study

**DOI:** 10.1186/s13104-022-06185-y

**Published:** 2022-09-06

**Authors:** Tanyanop Techasupaboon, Vasin Vasikasin, Narittaya Varothai, Navee Raknaisil, Worapong Nasomsong

**Affiliations:** 1 Department of Internal Medicine, Phramongkutklao Hospital and College of Medicine, Bangkok, Thailand; 2 Department of Internal Medicine, Ananda Mahidol Hospital, Lopburi, Thailand; 3 Division of Infectious Disease, Department of Internal Medicine, Phramongkutklao Hospital and College of Medicine, Bangkok, Thailand; 4 Division of Nephrology, Department of Internal Medicine, Phramongkutklao Hospital and College of Medicine, Bangkok, Thailand; 5 Department of Internal Medicine, Ananda Mahidol Hospital, Lopburi, Thailand; 6 Division of Infectious Disease, Department of Internal Medicine, Phramongkutklao Hospital and College of Medicine, Bangkok, 10400 Thailand

**Keywords:** End-stage chronic kidney disease, Hemodialysis, *S. aureus* nasal carriage, *S. aureus* bloodstream infection

## Abstract

**Objective:**

*Staphylococcus aureus* nasal carriage screening among hemodialysis patients is not standard practice in Thailand, because of data lacking regarding prevalence and correlation with subsequent infection. We aimed to investigate the prevalence of *S. aureus* nasal carriage and its association with bloodstream infection among hemodialysis patients. In this prospective multicenter cohort study, participants were screened for *S. aureus* nasal carriage over 2 consecutive weeks. Incidence of *S. aureus* bloodstream infection over the next 12 months was observed.

**Results:**

The prevalence of *S. aureus* nasal carriage was 11.67%. Incidence of *S. aureus* bacteremia among participants with and without *S. aureus* nasal carriage were 7.1% and 3.8%, respectively. The odds ratio for nasal carriage and subsequent bacteremia was 1.96 (95% CI 0.04–21.79; p = 0.553). Survival analysis showed that time to bacteremia among participants in the two groups did not significantly differ (p = 0.531). Prevalence of *S. aureus* nasal carriage among hemodialysis patients in Thailand was low. Patients presenting with *S. aureus* nasal carriage did not have increased risk of *S. aureus* bacteremia after 12-month follow-up. Nasal *S. aureus* screening and decolonization should not be encouraged in this setting.

**Supplementary Information:**

The online version contains supplementary material available at 10.1186/s13104-022-06185-y.

## Introduction

Bloodstream infection is the most common type of infection among hemodialysis patients, with *Staphylococcus aureus* as the main causative pathogen [1]. *S. aureus* colonizes humans especially in the nasal area, and carriage is recognized as an important risk factor for subsequent bloodstream infection [2].

Hemodialysis patients appear to have a greater risk of *S. aureus* colonization. An observational study reported a high rate of *S. aureus* nasal carriage among hemodialysis patients (40%), compared to normal populations (27%) [3]. An artificial nasal colonization study found that the average duration of *S. aureus* colonization was 4 days in the non-carriage group, and 14 days in the intermittent *S. aureus* nasal carriage group. However, among patients with persistent *S. aureus* nasal carriage, duration of colonization has been observed for up to 157 days [4].

Screening of *S. aureus* nasal carriage among hemodialysis patients and decolonization using mupirocin ointments exhibited more than 80% reduction in *S. aureus* infection and bacteremia [5]. Therefore, screening for *S. aureus* nasal carriage among hemodialysis patients is a routine practice in several countries [6, 7].

In Thailand, community-acquired methicillin resistant *S. aureus* (CA-MRSA) has not been observed and there is a lack of data regarding the prevalence of nasal carriage and association with invasive diseases [8]. Given the difference in epidemiology, screening of *S. aureus* nasal carriage is not a standard practice in Thailand. We therefore aimed to investigate the prevalence of *S. aureus* nasal carriage and its association with *S. aureus* bloodstream infection among hemodialysis patients.

## Main text

### Material and methods

#### Study setting and design

We conducted a prospective multicenter cohort study. Patients with end-stage renal disease (ESRD) who received hemodialysis at Phramongkutklao Hospital and Ananda Mahidol Hospital hemodialysis centers from July 2020 to September 2020 were enrolled. We estimated a sample size of 145 participants based on a previous study showing *S. aureus* nasal carriage among 40% of ESRD patients [3]. This provided 80% power to demonstrate the correlation between *S. aureus* nasal carriage and *S. aureus* bacteremia, with a two-sided alpha level of 0.05. The inclusion criteria included: age over 18 years, diagnosis of ESRD, receiving conventional hemodialysis, and regular follow up at two study hospitals. We excluded any patients who were undergoing hemodialysis for acute kidney injury.

#### Nose swab and bacterial isolates

All patients completed study questionnaires regarding their sociodemographic data and medical history. Bacterial swabs were obtained by study staff from the nasal mucosa at anterior nares once a week for 2 consecutive weeks. On each occasion, the cotton tip was inserted 1 cm into one nostril, rotated four times with slight pressure against the nasal septum, and transported with Amies transport medium. Each specimen was gently rolled and streaked on 5% sheep blood agar (BA) which were incubated at 37 °C for up to 24 h. Identification of *S. aureus* was carried out following standard microbiologic methods recommended by Clinical and Laboratory Standards Institute (CLSI) guidelines [9]. Positive swabs were defined as a colony forming unit (CFU) of > 10^3^. *S. aureus* isolates were inoculated in Mueller Hinton Agar and methicillin susceptibility was identified by disc diffusion method using 30 µg cefoxitin disc. Isolates with zone diameters of  ≥ 25 mm were classified as methicillin-susceptible whereas zone diameters of ≤  24 mm were classified as resistant, according to CLSI guidelines [9].

#### *Staphylococcus aureus* nasal carriage and *S. aureus* bacteremia

Participants were classified into three groups based on their *S. aureus* nasal carriage: no nasal carriage, intermittent nasal carriage, and persistent nasal carriage. Intermittent nasal carriage was defined as a positive nasal swab in only one of the two specimens, while persistent nasal carriage was defined as positive nasal swabs in both specimens [10, 11]. All participants were followed up until 12 months after the last nasal swab collection for their status of *S. aureus* bacteremia, defined as at least one isolation of *S. aureus* from a blood culture. Participants lost to follow up were excluded from the analysis but were interviewed by phone for the admission with signs or symptoms compatible with bacteremia. Participants and primary doctors were not informed of the carrier status and all participants presenting *S. aureus* nasal carriage did not receive any decolonization intervention.

#### Statistical analysis

Baseline characteristics were analyzed using descriptive statistics. Categorical data were presented as percentages while mean and standard deviation (SD) were used for continuous data. For categorical variables, Fisher’s exact test or the Chi-square test was used, while Mann–Whitney test or Student’s T-test was used to compare continuous variables. The relationship between *S. aureus* nasal carriage and *S. aureus* bloodstream infection was analyzed using odds ratios and was presented with a 95% confidence interval. We used Kaplan–Meier method to estimate the cumulative bacteremia and stratified log-rank statistic to assess the risk of nasal carriage compared with non-nasal carriage. For all analyses, a two-sided p-value of 0.05 was considered significant. All statistical analyses were performed using Stata 12.0 Software (StataCorp, USA).

## Result

From July 2020 to September 2020, 141 hemodialysis patients at the two hemodialysis centers were enrolled. Nine participants declined to participate in the study and 12 participants were excluded owing to changes in their hemodialysis center during this period. These 12 participants were all non-carriers. They did not report admission with signs or symptoms compatible with bacteremia upon phone interview. Hence, we included 120 participants (Additional file1: Fig S1).

### Patient characteristics

Of 120 participants, the mean age was 54.9 ± 16.07 years. Half of the participants were female. The two most common routes of hemodialysis were arteriovenous fistula, and tunneled hemodialysis catheter, respectively. The most common underlying diseases were hypertension, dyslipidemia, and diabetes. Female sex and dyslipidemia were significantly more common in the carrier group (OR 4.11; 95%CI 1.08–15.56, p = 0.027, and OR 4.60; 95%CI 1.21–17.45, p = 0.008 respectively). Baseline characteristics are described in Table 1.

**Table 1:** Baseline characteristics of hemodialysis patients presenting *S. aureus* nasal carrier and non-carrier

Characteristic	Carrier (n = 14) n (%)|Mean ± SD	Noncarrier (n = 106) n (%)|Mean ± SD	Total (n = 120) n (%)|Mean ± SD	p-value
Age	55.14 ± 17.15	54.89 ± 16.10	54.9 ± 16.07	0.959
Female	11(78.57%)	50(47.17%)	61(50.83%)	0.027
BMI	22.01 ± 6.12	23 ± 4.32	21.4 ± 4.57	0.566
Route of hemodialysis				
Double lumen catheter	1(7.14%)	5(4.72%)	6(5%)	0.696
Tunneled hemodialysis catheter	5(35.71%)	35(33.02%)	38(31.67%)	0.841
AVF	6(42.86%)	57(53.77%)	63(52.5%)	0.442
AVG	2(14.29%)	9(8.49%)	11(9.17%)	0.480
Prior antibiotic	9(32.14%)	20(21.74%)	29 (24.17%)	0.260
Immunosuppressive use	1(7.14%)	8(7.54%)	9(7.5%)	0.957
Underlying disease				
Hypertension	12(85.71%)	98(92.45%)	110(91.67%)	0.391
Diabetic	4(28.57%)	29(27.36%)	33(27.5%)	0.924
Dyslipidemia	11(78.57%)	47(44.34%)	58(48.33%)	0.008
SLE	1(7.14%)	3(2.83%)	4(3.33%)	0.398
Skin disease	1(7.14%)	5(4.72%)	6(5%)	0.696
Malignancy	1(3.57%)	2(2.17%)	3(2.5%)	0.678
Renal cell cancer	1(7.14%)	0	1(0.83%)	1
Prostatic cancer	0	2(1.89%)	2(1.67%)	1

*AVF* arteriovenous fistula, *AVG* arteriovenous Graf, *SLE* systemic lupus erythematous

The prevalence of *S. aureus* nasal carriage was 11.67% (14 /120), with ten cases classified as intermittent and four cases classified as persistent nasal carriage. The prevalence of *S. aureus* nasal carriage in hemodialysis patients of Phramongkutklao and Ananda Mahidol Hospital was 10.71% and 11.96%, respectively (p = 0.858). All *S. aureus* isolates were susceptible to methicillin (methicillin-susceptible *S. aureus*; MSSA).

*Staphylococcus aureus* bacteremia occurred in five participants after 12 month follow-up (incidence = 4.17%). Among participants with *S. aureus* nasal carriage, one participant in the intermittent nasal carriage group had *S. aureus* bacteremia (incidence = 7.1%; 1/14), while 4 participants in the non-nasal carriage group *had S. aureus* bacteremia (3.8%; 4/106). Participants with nasal carriage were not more likely to develop bacteremia than participants presenting non nasal carriage at 12 months (OR 1.96; p = 0.553).

**Fig. 1:**
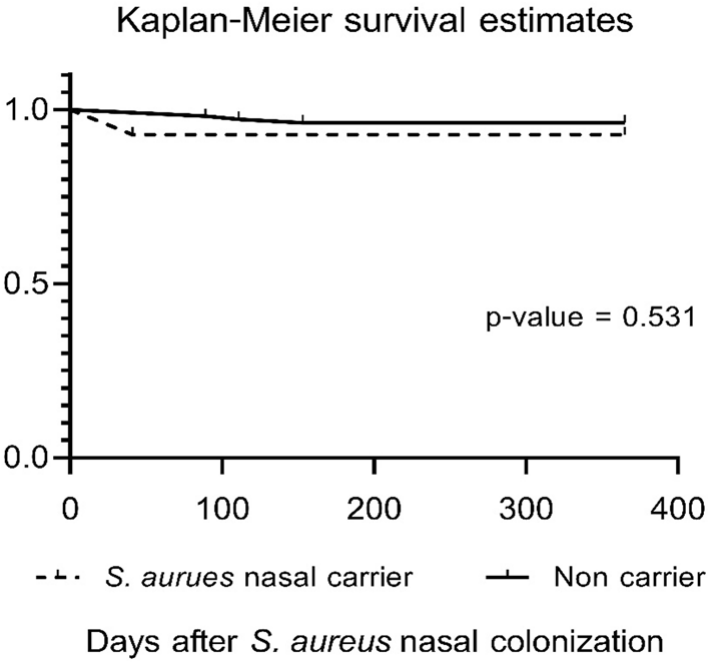
Kaplan Meier curve demonstrating cumulative *S. aureus* bacteremia among participants with and without nasal carriage

Clinical characteristics of participants presenting bacteremia are described in Table 2. All *S. aureus* isolated in blood culture were MSSA. Among participants presenting bacteremia, nasal carriage patients developed bacteremia in 41 days while non nasal carriage patients developed bacteremia at 89, 89, 111 and 153 days, respectively. Cumulative bacteremia did not significantly differ between the two groups (p = 0.531) (Fig. 1).

**Table 2:** Clinical characteristics of *S. aureus* bacteremia participants

No	Age/Sex	Vascular access	Nasal carriage status	Hemoculture (Bacteremia)	Clinical syndrome	Treatment/outcome
1	35/Female	DLC IJV	Intermittent	MSSA	CRBSI	IV Cloxacillin, removed catheter/ Cured
2	60/Male	Tunneled hemodialysis catheter	None	MSSA	CRBSI	IV Cloxacillin, removed catheter/ Cured
3	59/Male	Tunneled hemodialysis catheter	None	MSSA	CRBSI	IV Cloxacillin, removed catheter/ Cured
4	74/Female	Tunneled hemodialysis catheter	None	MSSA	CRBSI	IV Cefazolin, removed catheter / Cured
5	67/Male	AVG	None	MSSA	Infected AVG	IV Cefazolin, debridement/ Cured

*AVF* arteriovenous fistula, *AVG* arteriovenous graf, *DLC* double lumen catheter, *IJV* internal jugular vein, *MSSA* methicillin-susceptible *Staphylococcus aureus*, *CRBSI* catheter related blood stream infection

## Discussion

In this prospective multicenter cohort study among hemodialysis patients, the prevalence of *S. aureus* nasal carriage was 11.7%. This number was lower than reported in other studies. For example, a related systematic review and meta-analysis among 2,374 participants revealed the prevalence of *S. aureus* nasal carriage at 26% [5]. Another report also found a 40% prevalence of *S. aureus* nasal carriage among hemodialysis patients [3]. Several factors may have influenced this effect. Firstly, the prevalence of *S. aureus* nasal carriage in the general population in Thailand is low. Related studies in Thailand reported the prevalence of *S. aureus* nasal carriage at 20, 13.9, and 3.6% among patients with allergic rhinitis, patients undergoing elective cardiac surgery, and pre-admission screening, respectively [12–14]. These proportions are lower than the reported prevalence in the US and Germany at 22% and 40%, respectively [4, 15]. Although the prevalence of nasal carriage among hemodialysis patients is usually higher when compared with the general population [3], we found that the prevalence of carriage in our cohort was close to that of the general Thai population. This also correlated with the lower incidence of *S. aureus* infection in hospitals and the lower colonization rate among health care personnel in Thailand [13–17]. Secondly, in this present study, most participants had permanent vascular access. This was associated with a lower risk of *S. aureus* nasal carriage compared with temporary dialysis access [18]. Finally, the prevalence of *S. aureus* nasal carriage may depend on several factors particularly, ethnicity, previous antibiotic use, socioeconomic status, and geographic differences between countries, as well as personal hygiene [18, 19].

The present study describes female sex and dyslipidemia being associated with *S. aureus* carriage. Association between sex and *S. aureus* nasal carriage status remains controversial [20, 21]. Women with low levels of circulating testosterone may have increased probability of persistent *S. aureus* carriage [22]. The nasal colonization of *S. aureus* among patients undergoing hemodialysis depends on underlying host conditions especially skin disease and diabetic mellitus. Nevertheless, dyslipidemia has not been established as a risk factor in related studies [23]. However, the association that we found in this present study may not represent the true relationship, because other confounding variables were not explored or controlled for. Well-designed studies are warranted to resolve this situation.

This study did not find any association between *S. aureus* nasal carriage and bacteremia. This may be explained by the unexpectedly low prevalence of *S. aureus* nasal carriage, compared with the prevalence at 40–65% reported in several studies which described associations between nasal carriage and bacteremia [3, 10, 24]. However, the rate of invasive infection within 6 to 20 months of nasal carriage was 19% [25], much higher than the 4.17% found in this study. Therefore, another possible explanation is the difference in the virulence of *S. aureus* between the studies. Although this study was conducted in tertiary hospital hemodialysis centers, we did not find MRSA. This conforms with reported data in Thailand which indicated an extremely low rate of MRSA nasal colonization either among patients or healthcare personnel [13, 14, 17]. The molecular basis and biofilm-forming capacity of *S. aureus* in each strain play a major role in the ability to colonize and develop invasive diseases [2, 26–28]. Molecular characterization as well as biofilm production of the isolated *S. aureus* nasal carriage strain should be further investigated.

To our knowledge, this study constitutes the first report of the prevalence of *S. aureus* nasal carriers and the relationship between nasal carriage and *S. aureus* bloodstream infection among hemodialysis patients in Thailand.

## Conclusion

Prevalence of *S. aureus* nasal carriage among hemodialysis patients at two Thai tertiary hospitals was 11.67%. Patients who had *S. aureus* nasal carriage did not have increased risk of *S. aureus* bacteremia after 12 month follow-up. In healthcare centers with low *S. aureus* nasal carriage prevalence, nasal *S. aureus* screening and decolonization should not be encouraged.

## Limitations

First, some participants were lost to follow up, which might have influenced the study result. However, this subset of patients were all non-carriers and did not report admission with signs or symptoms compatible with bacteremia. Therefore, the true incidence of bacteremia of the non-carrier group, but not in carrier group, might be underreported. Second, the molecular characteristics of the *S. aureus* isolates were not identified. Therefore, we could not confirm the linkage between colonization and bacteremia. Nevertheless, only one patient was found in this group. Finally, the adherence to basic infection prevention strategies among hemodialysis centers was not monitored. Therefore, we could not ensure that the lower rate of *S. aureus* nasal colonization and infection was due to good adherence to basic infection control. However, no difference was found in colonization between the two study sites.

## Supplementary Information

**Additional file 1: Figure S1.** Participant enrollment.

## Data Availability

Data sharing is not applicable to this article as no datasets were generated or analyzed during the current study.

## Abbreviations

AVF: Arteriovenous fistula
AVG: Arteriovenous graft
DLC: Double lumen catheter
CKD: Chronic kidney disease
RRT: Renal replacement therapy
*S. aureus*: *Staphylococcus aureus*
ESRD: End stage renal disease
CA: Cancer
RCC: Renal cell carcinoma
HT: Hypertension
DLP: Dyslipidemia
SLE: Systemic lupus erythematous
MRSA: Methicillin-resistant *Staphylococcus aureus*
MSSA: Methicillin-susceptible *Staphylococcus aureus*
CRBSI: Catheter related blood stream infection
