# Supplementary figures and images for: Staphylococcus aureus nasal carriage and bloodstream infection among conventional hemodialysis patients in Thailand: a prospective multicenter cohort study
Source: BMC Res Notes. 2022 Sep 6;15:290. doi: 10.1186/s13104-022-06185-y (PMC9450322; doi:10.1186/s13104-022-06185-y)

**Additional file 1 Figure S1:** Participant Enrollment


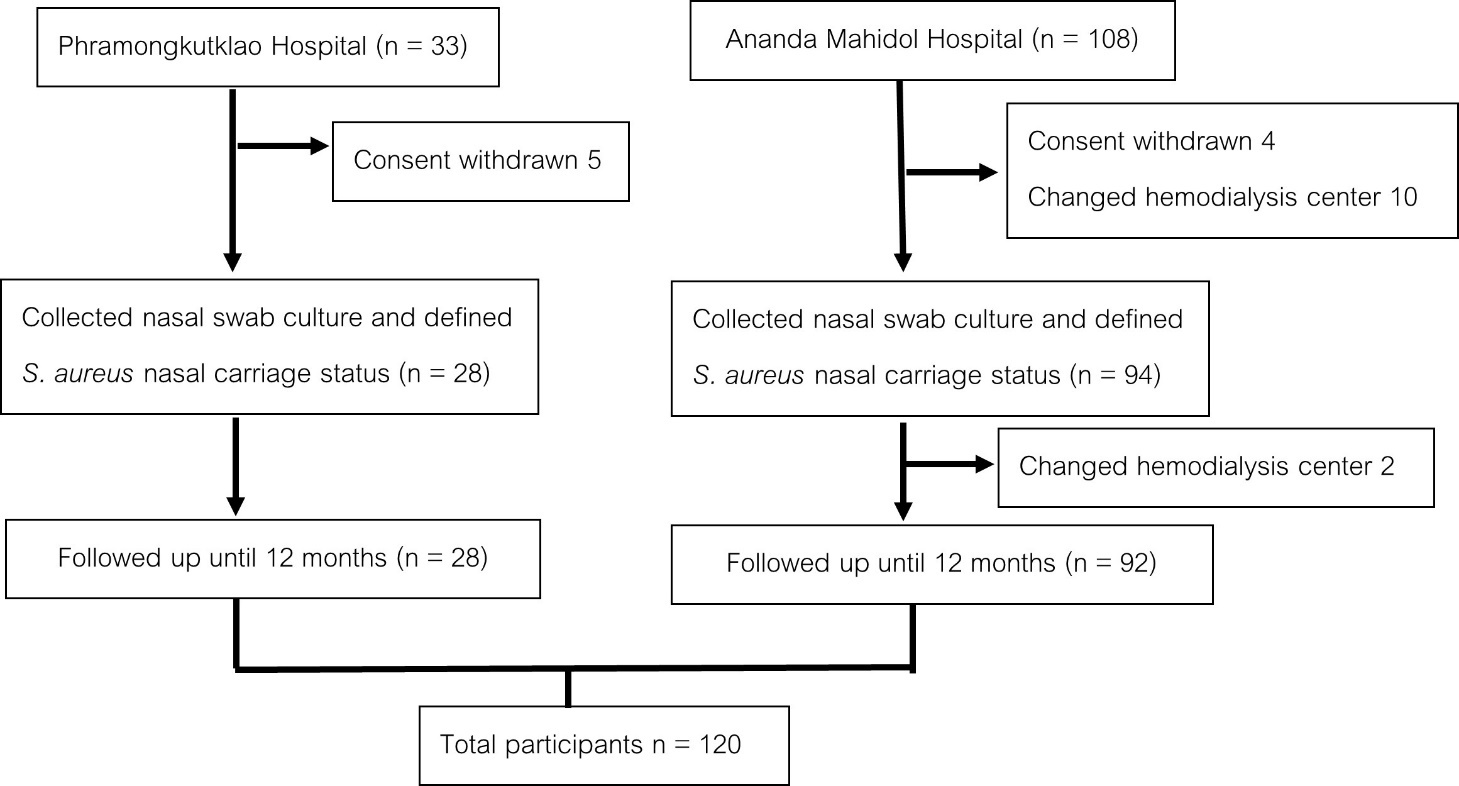

Supplement: Supplementary file 1 — Additional file 1: Figure S1. Participant enrollment. [file 13104_2022_6185_MOESM1_ESM.docx]
